# Supported Lipid Bilayer Platform for Characterizing the Membrane-Disruptive Behaviors of Triton X-100 and Potential Detergent Replacements

**DOI:** 10.3390/ijms23020869

**Published:** 2022-01-14

**Authors:** Negin Gooran, Bo Kyeong Yoon, Joshua A. Jackman

**Affiliations:** 1 School of Chemical Engineering and Translational Nanobioscience Research Center, Sungkyunkwan University, Suwon 16419, Korea; negingrn@g.skku.edu; 2 School of Healthcare and Biomedical Engineering, Chonnam National University, Yeosu 59626, Korea

**Keywords:** virus inactivation, enveloped virus, Triton X-100, detergent, phospholipid membrane, supported lipid bilayer, quartz crystal microbalance-dissipation

## Abstract

Triton X-100 (TX-100) is a widely used detergent to prevent viral contamination of manufactured biologicals and biopharmaceuticals, and acts by disrupting membrane-enveloped virus particles. However, environmental concerns about ecotoxic byproducts are leading to TX-100 phase out and there is an outstanding need to identify functionally equivalent detergents that can potentially replace TX-100. To date, a few detergent candidates have been identified based on viral inactivation studies, while direct mechanistic comparison of TX-100 and potential replacements from a biophysical interaction perspective is warranted. Herein, we employed a supported lipid bilayer (SLB) platform to comparatively evaluate the membrane-disruptive properties of TX-100 and a potential replacement, Simulsol SL 11W (SL-11W), and identified key mechanistic differences in terms of how the two detergents interact with phospholipid membranes. Quartz crystal microbalance-dissipation (QCM-D) measurements revealed that TX-100 was more potent and induced rapid, irreversible, and complete membrane solubilization, whereas SL-11W caused more gradual, reversible membrane budding and did not induce extensive membrane solubilization. The results further demonstrated that TX-100 and SL-11W both exhibit concentration-dependent interaction behaviors and were only active at or above their respective critical micelle concentration (CMC) values. Collectively, our findings demonstrate that TX-100 and SL-11W have distinct membrane-disruptive effects in terms of potency, mechanism of action, and interaction kinetics, and the SLB platform approach can support the development of biophysical assays to efficiently test potential TX-100 replacements.

## 1. Introduction

Preventing viral contamination of biologicals and biopharmaceuticals produced by mammalian cell culture platforms is a major industry concern [1,2]. A wide range of virus purification technologies have been incorporated into manufacturing processes by virtue of removing virus particles through filtration and inactivating virus particles with heat, low pH, or solvents/detergents [3,4,5]. For inactivating membrane-enveloped viruses, one of the most effective and broadly useful methods has involved detergents such as Triton X-100 (TX-100), which can disrupt the viral membrane envelope and hence abrogate viral infectivity. TX-100 is widely used for virus inactivation in the bio-manufacturing industry because it is generally regarded as safe, has acceptable product compatibility, is fast acting, and can be readily incorporated into manufacturing processes [4,5]. However, a phenol-containing metabolic product of TX-100 is a known endocrine disruptor that is toxic to aquatic organisms [6], which has spurred recent legislation in the European Union that will lead to phasing out TX-100 from industry usage [7,8].

This issue has led biopharmaceutical manufacturers to search for more environmentally friendly detergents that can replace TX-100 while displaying functionally equivalent virus inactivation properties [9,10,11]. Accordingly, to test detergent candidates, the major focus has been on treating selected membrane-enveloped viruses with different detergent concentrations under varying environmental conditions and exposure times and measuring the resulting effects on viral infectivity. This experimental approach has led to identifying several detergents with potentially suitable virus-inactivating properties, including the following two compounds: (1) Simulsol SL 11W (SL-11W), which is a commercially available glycoside surfactant that consists of an 11-carbon long, fatty alcohol joined together with a glucose molecule [12]; and (2) Nereid, which is a recently developed, phenol-free detergent that is structurally similar to TX-100 and not yet commercially available [13]. Depending on the study, the detergent candidates have either been tested alone or in direct comparison with TX-100, and the quantitative basis for performance evaluation has been the magnitude of the drop in viral infectivity and the corresponding inactivation kinetics. While viral infectivity measurements will remain an important component of detergent evaluation for this application and bacteriophage-based viral surrogates [14] are being developed in this direction, the experimental readouts mainly focus on biological effects and it is also critical to understand the biophysical mechanism of action of how these detergents work, especially in comparison to TX-100.

For example, TX-100 inhibits membrane-enveloped viruses [15,16,17,18] and causes membrane solubilization [19,20], while SL-11W has also been reported to inhibit membrane-enveloped viruses and is speculated, but not proven, to cause membrane solubilization [12]. It is important to investigate such mechanistic possibilities while more broadly developing experimental capabilities that can evaluate prospectively the membrane-disruptive properties of detergent candidates to replace TX-100. Indeed, recent experimental efforts utilizing membrane-mimicking supported lipid bilayer (SLB) platforms have helped to clarify the biophysical mechanism of action and potency of other membrane-disruptive detergents such as medium-chain fatty acids and monoglycerides, which correlated well with antiviral mitigation properties [21,22,23]. Translating such predictive measurement capabilities to support the development of detergents to replace TX-100 could be useful in terms of accelerating testing speed, reducing costs, and increasing lab safety as well as in providing a biophysical framework to build structure–function relationships and guide structural optimization [24].

Towards this goal, herein, we comparatively evaluated the membrane-disruptive properties of TX-100 and SL-11W against SLB platforms and identified that the two compounds interact with model phospholipid membranes in distinct ways. We first performed solution-phase fluorescence spectroscopy measurements to determine the critical micelle concentration (CMC) values of TX-100 and SL-11W since most membrane-disruptive surfactants are mainly active at and above their respective CMC values. These efforts were followed by quartz crystal microbalance-dissipation (QCM-D) experiments, in which we added different bulk concentrations of TX-100 or SL-11W to a phospholipid-based SLB platform and tracked corresponding, time-resolved changes in the mass and viscoelastic properties of the SLB platform due to detergent-induced membrane disruption. This combined experimental approach enabled us to compare the membrane-disruptive potency and mechanism of action of TX-100 and SL-11W and to postulate working models of how each compound interacts with phospholipid membranes. The real-time measurement capabilities and fast response time afforded by utilizing the SLB platform in conjunction with the QCM-D technique further demonstrate the potential for establishing rapid biophysical assays that could complement the current industry focus on biological assays measuring viral inactivation parameters.

## 2. Results and Discussion

### 2.1. Micellar Aggregation Properties

TX-100 is a nonionic detergent that consists of a hydrophilic polyethylene oxide chain with nine to ten ethylene oxide units, on average, and a hydrophobic 4-(1,1,3,3-tetramethylbutyl)-phenyl group. SL-11W is also a nonionic detergent and has a hydrophilic glucose headgroup that is connected to a hydrophobic 11-carbon long, fatty alcohol tail. Since TX-100 and SL-11W are single-chain, amphipathic detergent molecules, they both have a propensity to self-assemble in aqueous solutions, which is an important characteristic that can influence membrane-disruptive properties [25,26].

**Figure 1:**
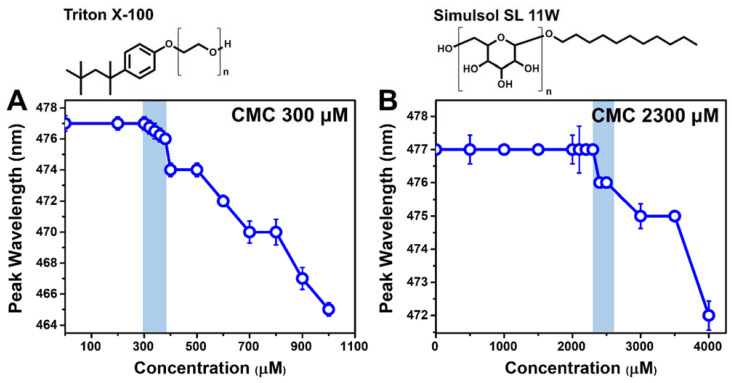
Critical micelle concentration (CMC) values for TX-100 and SL-11W based on fluorescence spectroscopy measurements. Peak wavelength of fluorescent probe (1-pyrenecarboxaldehyde) as a function of test compound concentration in PBS for (**A**) TX-100 and (**B**) SL-11W. The chemical structure of each detergent molecule is presented above the corresponding graph. The n values for TX-100 and SL-11W are 9–10 and 1–1.25, respectively, and were defined accordingly to be 9.5 and 1 for molecular weight calculations. Each data point represents the mean ± standard deviation from four technical replicates. The highest compound concentration at which there is no peak shift (relative to the baseline) is defined as the CMC value and indicated by shading.

We proceeded to measure the CMC value of each detergent in PBS by wavelength-shift fluorescence spectroscopy measurements (Figure 1). Different concentrations of TX-100 or SL-11W were mixed with the probe molecule, 1-pyrenecarboxaldehyde, which can partition into the hydrophobic interior of micelles and has distinct fluorescence emission properties in aqueous environments and in the micelle interior [27]. In PBS alone, the maximum-intensity emission (peak) wavelength of the probe was 477 nm and remained at this value in the presence of low concentrations of TX-100 or SL-11W. At the onset of micelle formation, i.e., the CMC, the peak wavelength of the probe decreased due to the change in local dielectric properties as probe molecules partitioned into the hydrophobic interior of micelles. The corresponding CMC values in PBS were determined to be around 300 and 2300 μM for TX-100 and SL-11W, respectively. The experimentally measured TX-100 CMC value agrees well with past reports [28,29], and the results indicate that TX-100 forms micelles at approximately seven-fold lower concentrations than SL-11W. The measured CMC values of TX-100 and SL-11W further allowed us to define the test concentration range (4000–125 μM in a two-fold dilution series) for the subsequent QCM-D experiments, which were directed at comparing the membrane-disruptive properties of the two detergents.

### 2.2. QCM-D Tracking of Detergent-SLB Interactions

The QCM-D technique was utilized to track the biophysical interactions between the detergent compounds and a supported lipid bilayer (SLB) platform. For characterizing membrane-disruptive compounds in a comparative fashion, there are several key measurement advantages of utilizing the SLB platform together with the QCM-D technique, including real-time measurement responses, short experimental duration, quantitative readouts, and well-controlled lipid bilayer properties. The two main measurement signals are the resonance frequency (∆f) and energy dissipation (∆D) shifts that correspond to changes in the hydrodynamically-coupled mass and viscoelastic properties of the adlayer, respectively, due to SLB formation and subsequent interaction steps [30]. Initially, the SLB was formed on a silica-coated sensor chip according to the bicelle method [31,32] and was composed of zwitterionic 1,2-dioleoyl-*sn*-glycero-3-phosphocholine (DOPC) lipids. The corresponding final ∆f and ∆D shifts were −24.6 ± 0.5 Hz and 0.16 ± 0.06 × 10^−6^, respectively, which indicate complete SLB formation [33]. Afterwards, different concentrations of TX-100 or SL-11W were added to the SLB platform under continuous flow conditions and the resulting ∆f and ∆D shifts were tracked as a function of time to characterize detergent-mediated membrane disruption, including membrane solubilization and/or membrane morphological changes depending on the detergent type and concentration.

#### 2.2.1. TX-100

**Figure 2:**
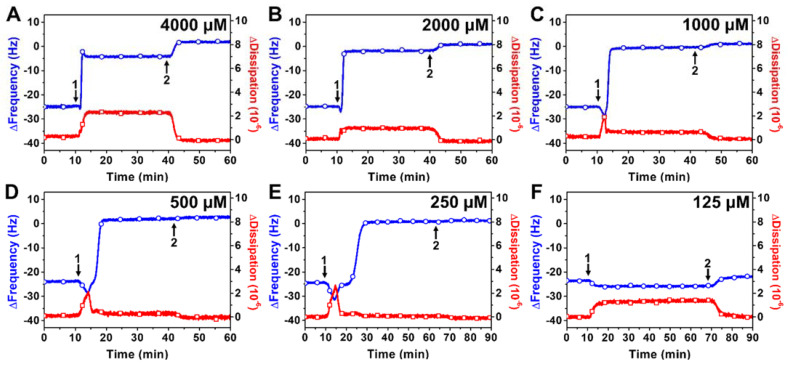
QCM-D tracking of the real-time interactions between TX-100 detergent and a DOPC SLB platform. Time-resolved ∆f (blue line with circles) and ∆D (red line with squares) shifts are presented as a function of time for SLB treatment with **(A)** 4000, (**B**) 2000, (**C**) 1000, (**D**) 500, (**E**) 250, or (**F**) 125 μM TX-100. The baseline values at *t* = 0 min correspond to a DOPC SLB adlayer and arrows 1 and 2 denote the start of compound addition and buffer washing, respectively.

Figure 2 presents representative time-resolved QCM-D measurement responses for the interaction of TX-100 with DOPC SLB platforms at different TX-100 concentrations. Upon treatment with 4000 or 2000 μM TX-100 concentrations, the ∆f shifts increased rapidly to around −2 to −5 Hz, indicating extensive loss of lipid mass due to membrane solubilization (Figure 2A,B). The ∆f shifts were accompanied by rapid ∆D shift increases to around 1–2 × 10^−6^, which remained stable. After a buffer washing step, the final ∆f and ∆D shifts were ~0 Hz and ~0 × 10^−6^, respectively, which support that high TX-100 concentrations caused complete membrane solubilization. Upon treatment with 1000, 500, or 250 μM TX-100 concentrations, there was an initial transient drop in the ∆f signal down to −30 to −35 Hz (~5–10 Hz magnitude) that lasted around 5–10 min and was accompanied by a transient increase in the ∆D signal up to ~2 × 10^−6^ (Figure 2C–E). The time scale of this transient interaction period was longer at lower TX-100 concentrations within this range, while the ∆f and ∆D shifts then proceeded to rapidly increase to ~0 Hz and decrease to ~0 × 10^−6^, respectively. A subsequent buffer washing step had negligible effect and complete membrane solubilization occurred in all cases.

In marked contrast, upon treatment with 125 μM TX-100 concentration, there was only a small drop in the ∆f signal down to −26 Hz (~2 Hz magnitude) and the ∆D shift increased to ~1 × 10^−6^ (Figure 2F). Upon buffer washing, the ∆f and ∆D shifts returned to nearly the baseline values of the complete SLB adlayer prior to detergent addition, indicating negligible membrane-disruptive effect. Altogether, the QCM-D data support that ≥250 μM TX-100 concentrations cause complete membrane solubilization that occurs through a fleeting, transient stage of membrane perturbation, while lower TX-100 concentrations do not disrupt SLB properties appreciably. Taking into account the concentration increments used with the different experimental techniques, this concentration-dependent trend is also consistent with the measured CMC value of TX-100 (~300 μM), supporting that micellar aggregation plays an important role in conferring membrane-disruptive properties.

#### 2.2.2. SL-11W

**Figure 3:**
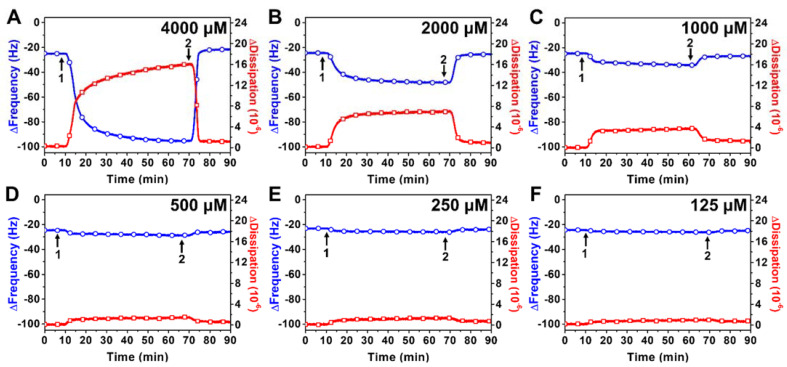
QCM-D tracking of the real-time interactions between SL-11W detergent and a DOPC SLB platform. Time-resolved ∆f (blue line with circles) and ∆D (red line with squares) shifts are presented as a function of time for SLB treatment with (**A**) 4000, (**B**) 2000, (**C**) 1000, (**D**) 500, (**E**) 250, or (**F**) 125 μM SL-11W. The baseline values at *t* = 0 min correspond to a DOPC SLB adlayer and arrows 1 and 2 denote the start of compound addition and buffer washing, respectively.

Figure 3 presents representative time-resolved QCM-D measurement responses for the interaction of SL-11W with DOPC SLB platforms at different SL-11W concentrations. Upon treatment with 4000 μM SL-11W concentration, the ∆f shift decreased gradually to around −90 Hz over approximately 50 min, which was mirrored by a ∆D shift increase to around 16 × 10^−6^ (Figure 3A). After buffer washing, the ∆f and ∆D shifts quickly reverted back to around the baseline values, indicating that the pronounced membrane morphological changes due to SL-11W treatment were reversible. The resulting membrane-disruptive effects were modest and did not involve membrane solubilization, which is distinct from the membrane-disruptive effects of TX-100. These QCM-D measurement responses bear resemblance to those of other nonionic surfactants, namely monoglycerides with 10- and 12-carbon long tails, and are suggestive of membrane budding events [21,34,35,36]. Upon treatment with 2000 μM SL-11W concentration, similar measurement responses occurred and the transient ∆f and ∆D shifts gradually reached around −45 Hz and 7 × 10^−6^, respectively (Figure 3B). Again, however, the final ∆f and ∆D shifts after buffer washing quickly returned to the baseline values, indicating that SL-11W did not exert appreciable membrane-disruptive effects or cause membrane solubilization.

On the other hand, upon treatment with 1000 μM SL-11W concentration, there were only relatively small, transient ∆f and ∆D shifts down to around −32 Hz (~7 Hz magnitude) and up to around 3 × 10^−6^, respectively (Figure 3C). After buffer washing, the final ∆f and ∆D shifts returned to the baseline values corresponding to a complete SLB adlayer. Upon treatment with 500, 250, or 125 μM SL-11W concentrations, there were negligible changes in the ∆f and ∆D shifts, indicating no membrane-disruptive effect (Figure 3D–F). Together, these results support that ≥2000 μM SL-11W concentrations cause extensive, budding-like membrane morphological changes in a reversible manner, demonstrating that SL-11W can interact with lipid membranes but does not cause membrane solubilization or permanent membrane disruption. Furthermore, the QCM-D data support that the membrane-perturbing effects of SL-11W also depend on micellar aggregation.

### 2.3. Comparison of TX-100 and SL-11W Membrane-Disruptive Effects

The QCM-D results demonstrate that TX-100 and SL-11W both interact with phospholipid membranes and cause membrane-disruptive effects, albeit in distinct ways. Indeed, while we classify both compounds as causing membrane disruption, which is defined as inducing irreversible or reversible changes in the mass and viscoelastic properties of the SLB platform as determined by the QCM-D measurements, the specific nature of membrane disruption caused by each compound is distinct. While the TX-100 interaction with model liposomal and biological membranes has previously been characterized using the QCM-D technique, incomplete membrane solubilization and/or other more complex measurement responses were observed in those past studies [37,38,39,40,41] and our findings demonstrate that the SLB platform combined with QCM-D measurement analysis is able to clearly detect complete membrane solubilization, i.e., full and irreversible removal of the adsorbed lipid bilayer mass from the sensor surface, which occurs on a short time scale, provided that the TX-100 concentration is around its CMC or higher. Determination of the lipid bilayer mass removal was judged by the final QCM-D measurement responses post-washing, which returned to the original baseline values prior to SLB fabrication when there was only buffer solution in the measurement chamber.

By contrast, the QCM-D results support that SL-11W is also a membrane-disruptive compound that can induce large-scale membrane morphological changes; however, the interaction process occurs more gradually than that of TX-100 and does not cause extensive membrane solubilization. Instead, in the SL-11W case, the transient QCM-D measurement shifts, namely the magnitudes of the ∆f and ∆D shifts, were suggestive of reversible membrane budding (see, e.g., data in refs. [21,34] for comparative examples), while the final QCM-D measurement responses post-washing supported that the SLB platform was mainly intact since the response values nearly corresponded to those of a complete SLB. Hence, our QCM-D measurement strategy aimed at tracking real-time biophysical interactions involving the SLB platform can elucidate the (1) concentration-dependent detergent potency, (2) biophysical mechanism of action related to detergent-mediated membrane disruption, and (3) timescale of membrane interaction processes.

**Figure 4:**
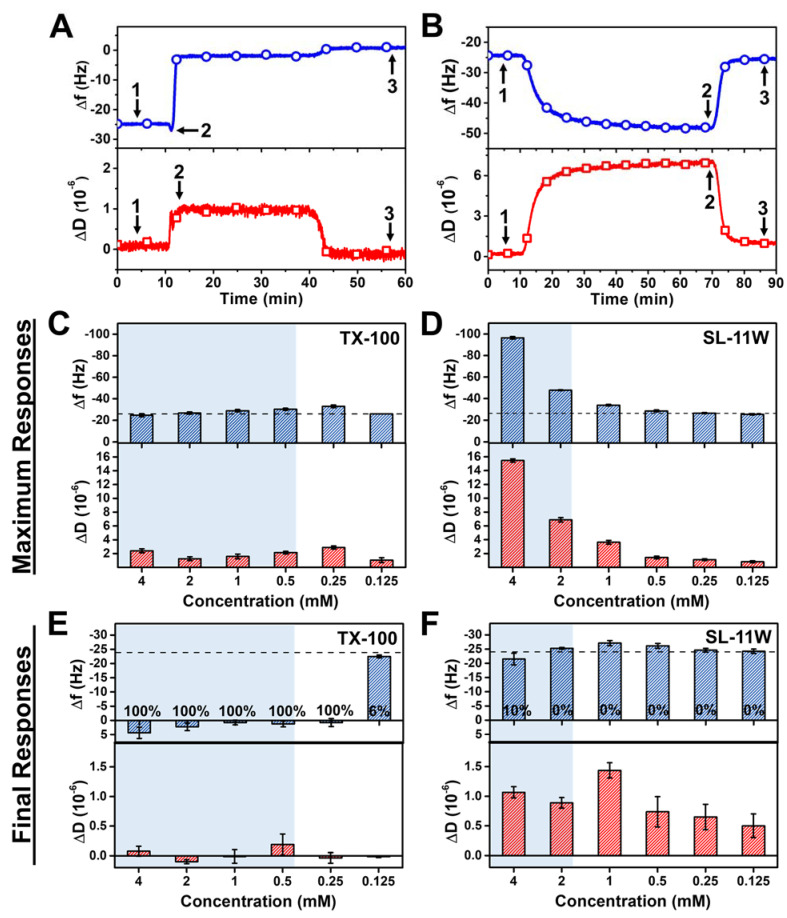
Comparison of QCM-D measurement responses for TX-100 and SL-11W interactions with SLB platform. Representative QCM-D measurement signals and corresponding data points that were used to define the baseline (arrow 1) and to calculate the maximum (arrow 2) and final (arrow 3) responses due to (**A**) TX-100 and (**B**) SL-11W detergent interactions with the SLB platform. Maximum ∆f (top panel) and ∆D (bottom panel) shifts corresponding to transient interaction processes occurring between (**C**) TX-100 or (**D**) SL-11W detergents and SLB platform. Final ∆f (top panel) and ∆D (bottom panel) shifts post-treatment corresponding to membrane-disruptive effects of (**E**) TX-100 and (**F**) SL-11W detergents on SLB platform after the last buffer washing step. The dashed lines denote the typical measurement values for a complete SLB adlayer. Shaded regions indicate concentration ranges in which the compounds are in the micellar state and the percentage values listed in (**E**,**F**) correspond to SLB removal fractions according to the Sauerbrey equation. Data are reported as mean ± standard deviation from four measurements.

Figure 4 presents a quantitative comparison of the membrane interaction processes for TX-100 and SL-11W based on the transient (i.e., maximum during interaction stage) and final QCM-D measurement responses post-washing. The quantification approach to determine these measurement shifts for each detergent case is shown in Figure 4A,B. For TX-100, the magnitudes of the transient ∆f and ∆D shifts were only minor, and the interaction process quickly led to complete membrane solubilization, as indicated by the QCM-D measurement shifts returning to baseline values that corresponded to no lipid bilayer mass on the sensor surface (Figure 4C). In marked contrast, for SL-11W, the transient, relatively large ∆f and ∆D shifts were appreciable and suggested detergent-mediated membrane budding that occurred to a greater extent at higher SL-11W concentrations, especially above its CMC value (Figure 4D). These trends support that TX-100 causes rapid membrane disruption that largely skips transient membrane interaction processes in terms of discernable measurement signatures, whereas SL-11W caused gradual membrane budding in a concentration-dependent manner that was distinct from the membrane-disruptive behavior of TX-100.

Furthermore, the final QCM-D measurement responses post-washing indicated distinct trends in membrane-disruptive behavior. For ≥250 μM TX-100 concentrations, the final QCM-D measurement responses corresponded to complete membrane solubilization, as indicated by final ∆f and ∆D shifts of around ~0 Hz and ~0 × 10^−6^, respectively, relative to the initial PBS baseline (prior to SLB formation) (Figure 4E). The extent of lipid bilayer removal, expressed in percentage units, was also estimated by converting the ∆f shift magnitudes into the corresponding adlayer mass values according to the Sauerbrey equation [42], which were then compared to the adlayer mass value of a complete SLB (~425 ng/cm^2^). In the aforementioned cases, ≥250 μM TX-100 induced ~100% lipid bilayer removal. On the other hand, for 125 μM TX-100 concentration, the final ∆f and ∆D shifts were around −24 Hz and ~0 ×10^−6^, respectively, which are equivalent to a nearly complete SLB adlayer (only ~6% lipid bilayer removal) and demonstrate that extensive membrane solubilization occurred in a CMC-dependent manner. Conversely, while SL-11W caused extensive membrane budding during the interaction process itself, the final ∆f and ∆D shifts were around −20 to −25 Hz and < 1 × 10^−6^, respectively, relative to the initial PBS baseline (prior to SLB formation) (Figure 4F). As such, SL-11W displays only moderate membrane-disruptive effects and does not cause extensive membrane solubilization (~10% lipid bilayer removal due to 4 mM SL-11W treatment and ~0% lipid bilayer removal at lower SL-11W concentrations), which supports that TX-100 and SL-11W exhibit distinct biophysical interactions with phospholipid membranes. It should be noted that the QCM-D measurements also support that TX-100 is fast-acting, whereas SL-11W induces transient membrane morphological changes more gradually.

**Figure 5:**
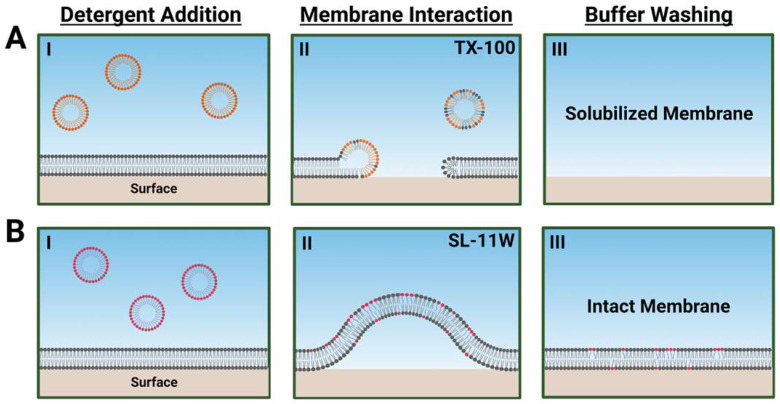
Schematic summary of TX-100 and SL-11W detergent interactions with DOCP SLB platform and proposed morphological changes based on QCM-D data. The DOPC phospholipid and TX-100 and SL-11W detergent molecules are represented by gray, orange, and magenta headgroup colors, respectively. Illustrative steps are shown for (**A**) TX-100 that induces rapid membrane solubilization starting from compound addition and resulting in complete lipid bilayer removal, and (**B**) SL-11W that induces membrane budding upon compound addition but does not cause membrane solubilization as indicated by final QCM-D measurement responses post-washing. Note that the spherical structures in AI and BI are intended to represent TX-100 and SL-11W detergent micelles, respectively, while the spherical structures in AII are intended to represent mixed micelles that form during membrane solubilization. In BII, membrane budding is driven by the intercalation of SL-11W molecules into the lipid bilayer, which causes local changes in spontaneous curvature.

Schematic illustrations describing how TX-100 and SL-11W detergents interact distinctly with phospholipid membranes, as proposed based on the QCM-D responses, are shown in Figure 5. The measurement data support that TX-100 causes rapid membrane solubilization, whereas SL-11W causes more gradual membrane budding in a transient manner and exhibits only modest membrane-disruptive effects after a buffer washing step. These findings demonstrate that TX-100 and SL-11W have different biophysical interactions with phospholipid membranes in terms of potency, mechanism of action, and interaction kinetics. While SL-11W has demonstrated virus-inactivating properties in bioreactor conditions, our biophysical findings suggest that TX-100 and SL-11W likely have distinct membrane-disruptive behaviors related to enveloped virus inactivation and direct comparison testing of SL-11W with TX-100 is warranted to validate its functional equivalence and further consideration as a potential TX-100 replacement.

## 3. Conclusions

In this study, we have employed the SLB platform as a biomimetic membrane tool to study the membrane-disruptive effects of TX-100 and SL-11W detergents from a biophysical perspective. By utilizing the QCM-D technique that is compatible with the SLB platform, the measurement results demonstrated that the two detergents have distinct membrane interactions in terms of potency, mechanism of action, and interaction kinetics. Of note, TX-100 solubilizes phospholipid membranes, whereas SL-11W does not solubilize phospholipid membranes. From a molecular perspective, the results further demonstrate that the two detergents were active in a CMC-dependent manner and that micellar aggregation plays an important role in conferring membrane-disruptive properties.

While viral inactivation studies are currently the main approach to evaluate detergent candidates to potentially replace TX-100, there has been recent discussion about how such approaches are costly, timely, and require high technical expertise and extensive safety considerations (see, e.g., ref. [14]). Hence, the development of more broadly accessible and rapid evaluation approaches would be advantageous. We envision that the SLB platform in conjunction with surface-sensitive measurement approaches such as the QCM-D technique could fill this measurement need by providing a well-controlled biomimetic membrane platform that is compositionally tunable, can be tested in various environmental conditions, and facilitates rapid screening in standard laboratory settings on the order of hours. 

Future improvements to the SLB platform might include using a membrane composition that is more representative of viral envelopes as well as exploring other types of model membrane platforms such as intact liposome adlayers that better mimic the membrane curvature of small, enveloped virus particles as opposed to the planar SLB configuration. Considering that viral envelopes also contain membrane-associated proteins, reconstitution of viral membrane extracts on sensor surfaces might also be considered (see, e.g., recent works involving other types of biological membranes [43,44]) and could be useful in terms of studying detergent interactions with a more complex milieu of lipids and proteins. In addition, while the QCM-D technique provides a useful label-free measurement approach for quantitative evaluation of detergent-mediated membrane disruption, deeper biophysical understanding of the corresponding interactions from a fundamental perspective could be obtained by utilizing additional techniques such as time-lapse fluorescence microscopy in conjunction with fluorescently labeled SLBs. As such, the SLB measurement capabilities and other related biomimetic possibilities could be useful to evaluate detergent candidates to replace TX-100 and provide a biophysical framework to optimize the molecular properties of virus-inactivating detergents.

## 4. Materials and Methods

### 4.1. Reagents

Triton X-100 (TX-100) and 1-pyrenecarboxaldehyde were purchased from Sigma-Aldrich (St. Louis, MO, USA) and Simulsol SL 11W (SL-11W) was obtained from Seppic Inc. (Fairfield, NJ, USA). 1,2-dioleoyl-*sn*-glycero-3-phosphocholine (DOPC) and 1,2-dihexanoyl-*sn*-glycero-3-phosphocholine (DHPC) lipids were acquired from Avanti Polar Lipids, Inc. (Alabaster, AL, USA). Phosphate-buffered saline (PBS, pH 7.5) was obtained from Gibco (Carlsbad, CA, USA). Milli-Q-treated water (>18 MΩ·cm) (Millipore, Billerica, MA, USA) was used for preparing all experimental samples.

### 4.2. Sample Preparation

Stock solutions of TX-100 and SL-11W were prepared by diluting the liquid-phase compounds in ethanol to a stock concentration of 200 mM. For experiments, the compounds were diluted 50-fold in PBS to the highest test concentration of 4 mM. After dilution, the samples were incubated in a water bath at 70 °C for 30 min to aid solubilization. Additional dilutions were made using PBS in a two-fold series.

### 4.3. Critical Micelle Concentration (CMC) Assay

The CMC values of TX-100 and SL-11W were determined by wavelength-shift spectroscopy measurements using a SpectraMax iD5 microplate reader (Molecular Devices, San Jose, CA, USA). A fluorescent molecule, 1-pyrenecarboxaldehyde, was used as the CMC probe and the excitation wavelength was set at 365 nm while the recorded emission wavelength range was 410 to 600 nm based on the probe properties, as previously described [34]. Briefly, a methanol stock solution of the probe was prepared at a 5 mM concentration and then aliquots were transferred to test vials, after which the vials were left in a fume hood for at least 30 min for solvent evaporation and drying. Then, the vials containing dry probe were hydrated in PBS solutions containing different concentrations of TX-100 or SL-11W and the final probe concentration was fixed at 0.1 μM. At least four technical replicates were measured per sample.

### 4.4. Quartz Crystal Microbalance-Dissipation (QCM-D) Measurements

The interactions between the test compounds, TX-100 and SL-11W, and the SLB platform were investigated by QCM-D measurements using a Q-Sense E4 instrument (Biolin Scientific AB, Gothenburg, Sweden), as previously described [21]. Briefly, the QCM-D technique tracks simultaneously time-resolved changes in the resonance frequency (∆f) and energy dissipation (∆D) signals of the piezoelectric sensor chip, and the ∆f and ∆D shifts correspond to the mass and viscoelastic properties of the SLB platform, respectively, on top of the sensor chip during SLB fabrication and due to resulting membrane morphological changes during subsequent protocol steps. Silica-coated QCM-D sensor chips (model No. QSX 303, Biolin Scientific AB, Gothenburg, Sweden) with a fundamental resonance frequency of 5 MHz were used and cleaned before each experiment as follows: (1) sequential rinsing with water and ethanol; (2) drying with a gentle stream of nitrogen gas delivered by a nozzle; and (3) removal of organic contaminants from the sensor surface by oxygen plasma treatment for 1 min using a CUTE-1MPR machine (Femto Science Inc., Hwaseong, Korea). After cleaning, the sensor chips were used immediately, and the initial measurement baseline was recorded in PBS. The compound addition and washing steps were all performed in PBS and all liquid samples were added under continuous flow conditions, as controlled by a peristaltic pump (Reglo Digital, Ismatec, Glattbrugg, Switzerland) that was set at a volumetric flow rate of 50 μL/min. The temperature in the measurement chamber was fixed at 25.0 ± 0.5 °C and the measurement data at the third, fifth, and seventh odd overtones were collected by the QSoft (Biolin Scientific AB, Gothenburg, Sweden) software package. Data processing was performed using the QTools (Biolin Scientific AB, Gothenburg, Sweden) and OriginPro 8.5 (OriginLab, Northampton, MA, USA) software packages and the reported data correspond to the fifth odd overtone and were normalized according to the overtone number.

## Data Availability

The data presented in this study are available upon reasonable request from the corresponding authors.
